# Determinants of caregiver's knowledge and practices regarding childhood fever management in a developing setting: a multi-centre cross-sectional assessment

**DOI:** 10.3389/fped.2023.1119067

**Published:** 2023-08-22

**Authors:** Ibrahim A. Ogunyinka, Kazeem A. Oshikoya, Kazeem B. Yusuff, Yusuf Tahir, Mohammed Yahaya, Sulaiman B. Adeniye, Innocent E. Oforkansi

**Affiliations:** ^1^ Department of Clinical Pharmacy and Pharmacy Practice, Faculty of Pharmaceutical Sciences, Usmanu Danfodiyo University, Sokoto, Nigeria; ^2^ Department of Pharmacology, Therapeutics and Toxicology, College of Medicine, Lagos State University, Ikeja, Nigeria; ^3^ Department of Clinical Pharmacy and Practice, College of Pharmacy, QU Health, Qatar University, Doha, Qatar; ^4^ Department of Paediatrics, Usmanu Danfodiyo University Teaching Hospital, Sokoto, Nigeria; ^5^ Department of Medical Microbiology and Parasitology, Usmanu Danfodiyo University, Sokoto, Nigeria; ^6^ Department of Research, Innovation and Development, Maributh Global Resources Limited, Sagamu, Nigeria

**Keywords:** adverse antipyretic-associated events, education, febrile convulsion, fever phobia, guidelines, nutritional status, pandemic, pre-hospital treatment

## Abstract

**Introduction:**

Fever is both a sign of various diseases (chief of which are infectious in nature) and an adverse effect of certain interventions (e.g. vaccines, drugs) in the pediatric population. It elicits anxiety among caregivers and healthcare professionals alike resulting in non-evidence based practices, adverse medication administration events, waste of scarce resources and overutilization of health facilities. The determinants of these practices among caregivers in the domiciliary contexts have not been well characterized in developing settings.

**Methods:**

We assessed the knowledge and practices of childhood fever and their determinants among caregivers in domiciliary settings in Northern Nigeria using a 41-item questionnaire between August 2020 and February 2021.

**Results:**

The questionnaire is reliable (knowledge: Cronbach's Alpha = 0.689; practice: Cronbach's Alpha = 0.814) and collected data on a total of 2,400 caregiver-child pairs, who participated in the study. Over two-third (68.3%; 1,640) of the caregivers expressed fever phobic tendencies. Paracetamol was the most commonly used medication and constituted 31.3% of medication administration adverse events reported by the caregivers. Only one out of every six knowledgeable caregivers engaged in evidence-based home childhood fever management practices (7% vs. 41.6%) with being a primary caregiver [Knowledge: odd ratio (OR): 2.81, 95% CI: 0.38; 5.68; *p* value: 0.04; Practice: OR: 1.65, 95% CI: 0.09; 7.33; 0.02] and having a child/children aged ≤3 years (knowledge: OR: 7.03, 95% CI: 4.89; 9.67, *p* value: 0.003; practice OR: 3.11, 95% CI: 1.27; 8.59, 0.007) determining both the knowledge and practices of childhood fever management in a household.

**Conclusions:**

The knowledge and practice of childhood fever management among caregivers were sub-optimal with being a primary caregiver and having a child/children aged ≤3 years being the significant determinants of each domain. These gaps underscore the dire need for targeted strategies aimed at improving childhood fever management by educating caregivers.

## Introduction

Childhood fever is a naturally conserved immune-protective mechanism aimed at preventing the febrile child from potentially harmful extraneous agents; conferring some survival advantages on the febrile child but occurring at a significant metabolic cost (1, 2). It involves a tightly coordinated rise in core body temperature in response to the presence of infectious diseases, inflammatory conditions, malignancy, immunization and following the administration of certain drugs (1, 3–5) and reportedly accounting for a third of all presenting complaints to pediatricians (6). In addition, fever-inducing diseases are the major underlying etiologies of morbidity and mortality in under-five children (7) and among 5–14 year olds globally (8).

Despite evidence to the contrary caregivers still consider childhood fever as a disease (and not as a symptom/sign of an underlying illness); making them to engage in non-evidence based practices (9, 10). This wrong perception of fever (that all fevers are harmful) among caregivers has been termed *fever phobia* or *fever anxiety* (11) or *fever turmoil* (2). This phobic behavior is pervasive globally among caregivers and healthcare-informed individuals (whether as professionals, parents or undergraduates) (12–17) and transcends race, cultures and whether an individual resides in a resource-rich country or not (3, 6, 18, 19). In addition, this behavior could lead to adverse medication administration events, waste of scarce resources and overutilization of the already overstretched health facilities (17, 20, 21).

Due to the ubiquitous nature of fever, it has been extensively studied with clinical practice guidelines (CPGs) (22) and expert consensus (16, 23) published on it; yet recommendation-practice gap persists even among healthcare providers. With regards to caregivers this gap leads to irrational use of antipyretics- such as alternating or combining antipyretic in a fever episode which may lead to confusion, dosing errors, toxicities and sometimes fatalities (11, 15, 20, 21, 24). The irrational use of antibiotics is also a common feature of this gap and may lead to dysbiosis with the attendant risks of antimicrobial resistance, antibiotic-associated diarrhea and late-onset dysbiosis-associated atopic and inflammatory diseases including diabetes (16, 21). Physical measures involving use of water on the febrile child is also a prevalent non-evidence based practice among caregivers (9, 25, 26); as it temporarily brings down the temperature modestly only for it to be increased again with associated increase in the child's discomfort. Another consequences of poor home management of childhood fever is late presentation of the febrile child and this could be fatal (27).

It is however essential to state that home management of fever using over-the-counter (OTC) antipyretics is an essential component of evidence-based home care therapeutics (22, 28). This concept has been promoted because of increasing patient: healthcare provider ratio and over-utilization of the overstretched healthcare facilities and resources (29, 30). Rational OTC antipyretic use in febrile children involves focusing on relieving their fever-associated discomfort or distress (such as observed pain, irritability, crying or stress) and general condition rather than reduction of the thermometer reading (22). This suggests that there is no need administering antipyretic medication to a child (above 3 months old) with high body temperature who displays no change in appetite, mood, behavior, motor activity, sleep-wake rhythm, daily habits and interest in playing.

The guideline recommendation of resorting to antipyretic pharmacotherapy only to relieve fever-associated discomfort or distress among febrile children requires knowledge of risk stratification among healthcare professionals (HCPs) (such as using “traffic light system”), community healthcare workforce and caregivers (using early warning signs learnt from the safety-netting advice provided by HCPs) so that febrile children at high risk will be easily identified and referred or presented early at healthcare facility for adequate and necessary attention. This knowledge of risk stratification becomes even more invaluable following the world re-opening and the resumption of international travels. Another example of risk stratification is to give priority to fever in children <3 months old as evidence exists that the height of their fever often equals the degree of severity of the underlying illness (16).

Cultural and regional variations have been reported to exist regarding the pre-hospital management of febrile children (10, 11, 18, 19, 31); with higher proportion of caregivers in developed settings likely to be more health-literate (like owning and using thermometers in determining a febrile child's body temperature, knowing the correct body temperature that defines fever in children, and less likely to use medicinal plants or present their febrile children/wards at patent medicine shops) than the caregivers in developing settings (9, 10, 16, 32). Systematic reviews and meta-analyses do not often include studies from developing settings (10) or include little of them (9, 30). In addition, a 2017 systematic review of CPGs on symptomatic management of childhood fever that included a CPG from South Africa (a developing country) found it to be the poorest in terms of its applicability and rated it low for rigor of development and editorial independence (although, it was eventually recommended for use with some modifications suggested) (33). Consequently, profile of the practices of caregivers of febrile children at the domiciliary settings of developing countries might not have been well characterized.

In addition, literature is scanty regarding the home care of febrile children in Northern Nigeria. We assessed the knowledge and practices of childhood fever and their determinants among caregivers in domiciliary settings in Northern Nigeria.

## Materials and methods

### Study setting and design

This was a cross-sectional, quantitative study that was conducted at the Departments of Pediatrics, Usmanu Danfodiyo University Teaching Hospital (UDUTH), and Specialist Hospital (SH), Sokoto. Between them, these hospitals offer services to well over 1.7 million pediatric patients annually (34). The study commenced in August 2020 and was completed in seven months.

### Study instrument

A 41-item questionnaire was developed after extensive search of relevant literature. Content validity of the questionnaire was assessed using five experts and the reliability (of the knowledge and practice items) analyzed using Cronbach's Alpha Coefficients and Intraclass Coefficients after conducting a pilot test on 20 caregivers, whose data were not included in the final analysis. The modifications required were minor and effected before the study proper commenced (questionnaire available as online Supplementary Material).

The resulting 41-item questionnaire comprised four sections: (A)- socio-demographics comprising 20 items that elicited data on caregiver-child pair, (B)- comprised four items related to knowledge and (C)- comprised 13 practice-related items and four items on adverse drug events experienced during medication administration.

### Sample size determination

From the sampling frame of 1.7 million pediatric patients annually (34) and using 7 months (August 2020 and February 2021), 50% for daytime patient population and 35% febrile children presenting at any health facility in Nigeria (35); 173,542 pediatric febrile patients now became the study population but only 3,429 (2.0%) caregivers were approached with 2,400 consenting to participate in the study.

### Data collection procedure

Following presentation of febrile children at the emergency pediatric unit (EPU) and pediatric outpatient clinic (POC), each eligible caregiver (those presenting with febrile children aged ≤17 during the study period) was approached and the study objectives were explained, this was reinforced by the use of a study information note that summarizes the study in a plain, easily understood language and consenting caregiver is required to sign on a consent form. Eligible caregivers that were not literate were briefed in any of the three most popular Nigerian languages (Hausa/Fulani, Igbo and Yoruba) and then required to thumbprint on the consent form if interested. All approached caregivers were informed that their refusal to participate in the study will not affect the quality of healthcare they will receive and that they are free to withdraw their consents anytime they see fit even while the interviews are underway. In all, 3,429 caregivers were approached and 2,400 of them consented to participate in the study.

The questionnaire was used to collect relevant information on the eligible and consenting child-caregiver pairs and their home healthcare delivery practices through an average of a 10 min face-to-face interviews that were conducted after the patients have been attended to by the physicians. Two trained research assistants were involved in the data collection in each study site.

### Operational definitions

In this study the temperature threshold for fever is axillary or tympanic temperature ≥38.0°C (35–37), and ≥39.5°C as temperature threshold for antipyretic interventions (15). Consequently, the febrile children's temperature at presentation were further categorized into ≤37.9°C (afebrile state), ≥38.0°C (febrile state) and ≥39.5°C (febrile state to initiate antipyresis).

Caregivers are those who help with the daily care activities of ill children (the care-recipients) often residing in the same home as the care-recipients (38). They include parents, siblings, relatives, friends, nannies and maids. The parents, siblings, relatives and friends belong to a category known as the informal caregivers while the paid help or hands whose primary responsibility in the home and the reason for their employment is to take care of ill children are known as the formal caregivers. They include nannies and nurses (38). In the case of febrile children/households with more than one caregiver, a primary caregiver refers to the main caregiver who responds to the febrile child's health-related needs or general well-being the most and is only represented by the second (ary) caregiver when the former is unavoidably absent from this duty/responsibility.

#### Data management outcome assessment/scoring methods

Regarding the four knowledge items; adequate knowledge includes cause of fever expressed as infectious, accuracy of presumptive diagnosis, perception that not all fevers are harmful and use of weight-based dosage schedules. With regards to the thirteen practice items; appropriate home treatment practices include given a febrile child plenty of fluid to drink; appropriate clothing-cover up when shivering and light clothing when sweating; not waking him/her up just to administer antipyretics; visited orthodox healthcare facility and used drugs as prescribed or recommended from these sources; use of manufacturer's measuring device for dosage measurement; eight-hourly administrations of paracetamol and ibuprofen; use of time on mobile phones, wrist watches and wall clocks to ensure accurate dosage interval; coaxing the febrile child via singing of lullaby, referring to the medication as candy, asking a loved one to administer the medication and praising and then rewarding the child after administration were used to ensure the child takes medication whenever required; not alternating nor combining two antipyretic medications during the fever episode; giving the same dose as the previous one immediately when the child vomits (or the caregiver skipped a dose) at least four hours before the next dose or gave the same dose but waited for the next dose as the vomiting (dose skipping) occurred close to the next dose and home storage of used medications in the refrigerator, out of children's reach and away from sunlight (15, 16). Adequate knowledge and appropriate practices were scored as +1 (for correct responses as highlighted above) and 0 for incorrect responses. Scores of ≥50% were designated as adequate knowledge and appropriate practices respectively (14).

Responding that all fevers are harmful and then listing the harmful consequences of untreated fever by caregivers was used to determine the fever phobic tendency of the cohort (11, 18, 25, 26, 30).

Evidence exists that some caregivers of febrile children may have adequate knowledge, yet engage in inappropriate practices (13); we then determined those with adequate knowledge that only engaged in appropriate practices of home-based care of their febrile children.

Nutritional status of the febrile children was assessed using undernutrition (underweight) according to the Indian Academy of Pediatrics classification (weight/age) as it includes ages 5 and above (39):

Grade I (mild)- 71%–80%.
Grade II (moderate)- 61%–70%.
Grade III (severe)- 51%–60%.
Grade IV (very severe)- ≤50%.
Normal weight- >80%.

Caregivers' socioeconomic status (SES) were classified as low, middle and high according to a previous study (40). SES was calculated by taking the average of the sum of the scores obtained from caregivers' educational level and occupation. When the adult involved is single, the denominator is 2 and 4 for currently married. Those with SES scores of <2.00 were classified as low, ≥2.00–<3. 00 as middle and ≥3.00 as high.

#### Statistical analysis

Data were analyzed using IBM SPSS statistics software, version 25.0 (IBM Corporation, Armonk, NY, USA: released 2017). Frequencies and proportions were used to describe categorical variables with means and standard deviations used for continuous variables. The sociodemographic profile of the caregiver-child pair such as socioeconomic status, presence of a second caregiver, coverage by National Health Insurance Scheme; and clinical variables such as nutritional status, history of febrile seizure were the independent variables. Knowledge and practice of home management of childhood fever were the dependent variables. Chi square statistics for independent samples was used to investigate the independent variables with statistically significant differences in proportions ((*p *≤ 0.05) and those found as such were then entered into a binary logistic regression model. The resulting odds ratio suggested the likelihood of determining/influencing the knowledge and practice of childhood fever management. The effect of potential confounders (such as place of residence, educational status, socioeconomic status, relationship with febrile child, etc) were adjusted for and treated as covariates. Effect size estimates were determined using Phi/Cramer's V Coefficient and interpreted by Cohen's criteria for statistically significant (*p *≤ 0.05) associations −0.01 (small effect); 0.30 (medium effect); 0.50 (large effect) (41). The values for the Phi and Cramer's V Coefficients produced by the Chi–square statistics from the data analysis of this study were the same except for occasional difference in signs (that is, +; −).

#### Ethics approval

The Health Research and Ethics Committees of Usmanu Danfodiyo University Teaching Hospital, and Specialist Hospital reviewed and approved the research protocol. In addition, there was strict adherence to Good Clinical Practice including observation of articles of Helsinki concerning biomedical research and human rights.

## Results

### Psychometric properties of the questionnaire

The questionnaire has a good internal consistency reliability for the knowledge and practice domains (knowledge: α = 0.689; 95% confidence interval-0.64; 0.72; practice: α = 0.814; 95% confidence interval-0.77; 0.88) and Intraclass Coefficients (knowledge: ICC = 0.677; 95% confidence interval-0.63; 0.75; practice: ICC = 0.798; 95% confidence interval-0.74; 0.92).

### Socio-demographic characteristics

A total of 2,400 caregiver-child pairs participated in the study and the response rate was 70.0% (2,400/3,429). The mean (±SD) age and range of the caregivers and their febrile children were 31.83 (±11.41) (95% CI: 28.62; 38.56) (18–69 years) and 3.70 (±3.77) (95% CI: 3.49; 3.91) (0.1–17 years) years respectively. The mean (±SD) weight and range of the febrile children are 12.90 (±7.41) (95% CI: 8.52; 13.96) (2.3–40 kg) kilogram. The children‘s mean (±SD) presenting temperature and range were 38.2 (±0.06)°C (95% CI: 36.79; 40.12) and 37.5–41.0°C respectively while their mean number and range in the households were 5 and 1–19 children respectively. Other sociodemographic information of the febrile children and their caregivers is given in Tables 1, 2 respectively.

**Table 1:** Sociodemographic characteristics of the 2,400 febrile children.

Age group (years)	*n* (%)
≤3	1,496 (62.3)
3.5–4.5	222 (9.3)
5–10	458 (19.1)
≥11	224 (9.3)
Gender	
Male	1,280 (53.3)
Female	1,120 (46.7)
Number of siblings (children)	
Only child	328 (13.7)
2–4	1,200 (50.0)
≥5	872 (36.3)
Child's position (*n* = 1,504)	
First child	496 (33.0)
Last child	1,008 (67.0)
Gender predominance	
Male	1,008 (42.0)
Female	672 (28.0)
Equal	504 (21.0)
Males only	124 (5.2)
Females only	92 (3.8)
Nutritional status	
Normal	540 (45.0)
Mild	696 (29.0)
Moderate	360 (15.0)
Severe	176 (7.3)
Very severe	88 (3.7)
Socioeconomic status	
Low	776 (32.3)
Middle	1,296 (54.0)
High	328 (13.7)
Temperatures at presentation (°C)	
≤37.9	792 (33.0)
38–39.4	1,256 (52.3)
≥39.5	352 (14.7)
History of febrile convulsion	
Yes	568 (23.7)
No	1,832 (76.3)
NHIS status	
Yes	112 (4.7)
No	2,288 (95.3)

NHIS, National Health Insurance Scheme.

**Table 2:** Sociodemographic characteristics of the 2,400 caregivers.

Age group (years)	*n* (%)
≤24	442 (18.4)
25–34	1,232 (51.3)
35–49	638 (26.6)
≥50	88 (3.7)
Gender	
Male	48 (2.0)
Female	2,352 (98.0)
Presence of second caregiver	
Yes	360 (15.0)
No	2,040 (85.0)
Relationship with child	
Parents	2,200 (91.7)
Grand parents	112 (4.7)
Other relatives	48 (2.0)
Older siblings	24 (1.0)
Friends	8 (0.3)
Others	8 (0.3)
Religion	
Islam	2,224 (92.4)
Christianity	176 (7.3)
Marital status	
Married	2,272 (94.7)
Divorced	80 (3.3)
Widowed	48 (2.0)
Type of current marriage (*n* = 2,272)	
Monogamy	1,648 (72.5)
Polygamy	624 (27.5)
Position in the polygamy (*n* = 624)	
First wife	176 (28.2)
Second wife	424 (67.9)
Third wife	16 (2.6)
Fourth wife	8 (1.3)
Tribe	
Hausa/Fulani	2,192 (91.3)
Yoruba	80 (3.3)
Igbo	104 (4.3)
Others	24 (1.0)
Place of residence	
Urban	1,848 (77.0)
Rural	552 (23.0)

### Fever phobia among study participants

Over two-third (68.3%; 1,640/2,400) of the caregivers responded that all fevers are harmful (that is, they demonstrated fever phobic tendencies) and listed the consequences of untreated fever as follows: dehydration (10.2%; 168/1,640), febrile convulsion (15.1%; 248), coma (7.3%; 120/1,640), paralysis (2.9%; 48/1,640), brain damage (2.4%; 40/1,640), death (36.6%; 600/1,640) and all of the above listed consequences (25.4%; 416/1,640).

### Body temperature of febrile children at presentation

**Figure 1:**
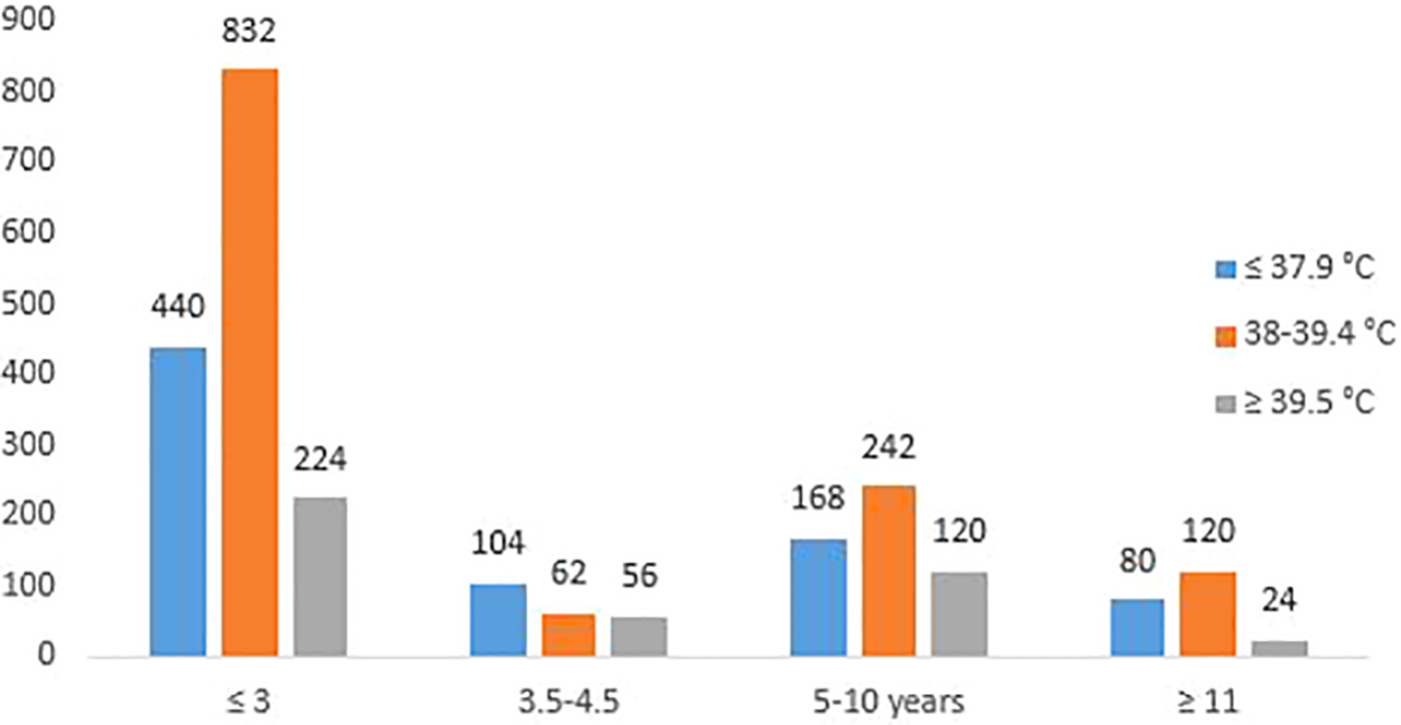
Shows the body temperatures of the 2,400 febrile children at presentation to the two study centres.

The youngest age group (those aged ≤3 years) significantly presented (at the centers) more than all other age groups (*p *= 0.047) during the study period (Figure 1). Similarly, they constituted the largest number of patients in each of the three temperature categories (≤37.9°C, 38.0–39.4°C, ≥39.5°C).

### Diagnoses of the febrile children

Caregivers' presumptive diagnoses and physicians' diagnoses were compared and only in 9.0% (216/2,400-all malaria-related) of the cases were caregivers' diagnoses accurate and it was statistically significant (*p *= 0.008).

### Caregivers' presumptive diagnoses

Malaria (25.7%; 616/2,400) topped the list of the presumptive diagnoses of the febrile children and closely followed by teething (24.0%; 576/2,400), change of weather (5.0%; 120/2,400), others (5.0%; 120/2,400) and most (40.3%; 968/2,400) of the study participants stated they could not presume.

### Physicians' diagnoses

Malaria of varying degree of severity constituted the highest number of diagnoses made. These were acute uncomplicated malaria (1,048; 43.7%), severe malaria (21.7%; 520/2,400) and cerebral malaria (10.3%; 248/2,400). Others were asthma (11.7%; 280/2,400), sickle cell diseases (8.7%; 208/2,400), bronchopneumonia (2.3%; 56/2,400) and measles (1.7%; 40/2,400).

### Home treatment of fever

Paracetamol (68.3%; 1,640/2,400) was the most commonly used medication during the pre-hospital management of the febrile children. Ibuprofen was the next most commonly used medication (44.9%; 1,078/2,400), followed by antimalarials (35.9%; 861/2,400) and antibiotics (32.2%; 773/2,400). The above medications were used singly or in combination.

### Use of antipyretics

Some (37.6%; 902/2,400) of the study participants reported combining paracetamol and ibuprofen during the home management of the current febrile episode and some of the reasons given for this inappropriate practice were: a HCP once prescribed/recommended it (62.1%; 560/902) and perceived lack of antipyretic response (body temperature was not being lowered quickly enough (24.3%; 219/902).

Those (14.8%; 355/2,400) who reportedly alternated between the use of paracetamol and ibuprofen while self-managing their febrile child/children at home stated running out of one of the antipyretic medications (31.8%; 113/355) and perceived lack of antipyretic response to the use of either of the antipyretics alone (20.8%; 74/355) as some of the reasons responsible for the non-evidence based practice.

### Adverse events from medication administration

Of the sixty-four study participants that reported medication administration-related adverse events, paracetamol use was the most culpable (31.3%; 20/64) followed by antibiotics (25.0%; 16/64), antimalarials (18.8%; 12/64) and cough syrups (12.5%; 8/64).

### Suspected adverse events and actions taken following the events

Vomiting and dizziness each reportedly accounted for 28 (43.8%; 28/64) of the medication administration-associated adverse events while forty caregivers (62.5%; 40/64) stopped the use of the suspected medication, eight (12.5%; 8/64).gave a lower dose and sixteen (25.0%; 16/64) continued the use.

### Knowledge and practice of childhood fever management among caregivers

Overall mean knowledge score of childhood fever was 1.66 (±0.22) (95% CI: 1.54; 1.76) and ranged between 0 and 4. Specifically, 41.5% (998/2,400) of the caregivers had adequate knowledge of childhood fever management.

Overall mean practice score of childhood fever was 4.69 (±0.91) (95% CI: 4.58; 4.79) and ranged between 0 and 9. Specifically, 7.0% (168/2,400) of the caregivers engaged in appropriate practice of childhood fever management.

### Based on characteristics of the febrile children

Table 3 showed the characteristics of the febrile children that has statistically significant differences in proportion with regard to knowledge and practice of home-based childhood fever management.

**Table 3:** Comparison of caregivers’ knowledge and practice of childhood fever management based on the sociodemographic characteristics of the febrile children (*N* = 2,400).

Characteristics	Knowledge inadequate	Adequate	*P* value	Practice inappropriate	Appropriate	*P* value
Age grp (yrs)						
≤3	855 (35.6)	641 (26.7)	0.862	1,368 (57.0)	128 (5.3)	**0.006**
3.5–4.5	143 (6.0)	79 (3.3)		206 (8.6)	16 (0.7)	
5–10	277 (11.5)	181 (7.5)		434 (18.1)	24 (1.0)	
≥11	134 (5.6)	90 (3.8)		224 (9.3)	0 (0)	
Total (%)	1,409 (58.7)	991 (41.3)		2,232 (93.0)	168 (7.0)	
Gender						
Male	654 (27.3)	626 (26.1)	**0.005**	1,224 (51.0)	56 (2.3)	**0.0001**
Female	738 (30.8)	382 (15.9)		1,008 (42.0)	112 (4.7)	
Total (%)	1,392 (58.1)	1,008 (42.0)		2,232 (93.0)	168 (7.0)	
Number of siblings (child.)						
Only child	141 (5.9)	187 (7.8)	**0.0005**	305 (12.7)	23 (1.0)	0.130
2–4	585 (24.4)	615 (25.6)		1,112 (46.3)	88 (3.7)	
≥5	595 (24.8)	277 (11.5)		836 (34.8)	36 (1.5)	
Total (%)	1,321 (55.0)	1,079 (45.0)		2,253 (93.8)	147 (6.2)	
Child's pos. *N* = 1,504						
First child	225 (15.0)	271 (18.0)	**0.00002**	472 (31.4)	24 (1.6)	0.108
Last child	514 (34.2)	494 (32.8)		920 (61.2)	88 (5.9)	
Total (%)	739 (49.2)	765 (50.8)		1,392 (92.6)	102 (7.5)	
Gender pred.						
Male	586 (24.4)	422 (17.6)	0.244	934 (38.9)	74 (3.1)	**0.004**
Female	403 (16.8)	269 (11.2)		647 (27.0)	25 (1.0)	
Equal	252 (10.5)	252 (10.5)		470 (19.6)	34 (1.4)	
Males only	89 (3.7)	35 (1.5)		107 (4.5)	17 (0.7)	
Females only	61 (2.5)	31 (1.3)		70 (2.9)	22 (0.9)	
Total (%)	1,391 (57.9)	1,009 (42.1)		2,228 (92.8)	172 (7.2)	
Nutritional status						
Normal	264 (1.0)	276 (11.5)	**0.000003**	500 (20.8)	40 (1.7)	0.09
Mild	532 (22.2)	164 (6.8)		648 (27.0)	48 (2.0)	
Moderate	264 (11.0)	96 (4.0)		328 (13.7)	32 (1.3)	
Severe	44 (1.8)	132 (5.5)		176 (7.3)	0 (0)	
Very severe	66 (2.8)	22 (0.9)		80 (3.3)	8 (0.3)	
Total (%)	1,710 (71.3)	690 (28.7)		1,732 (72.2)	668 (27.8)	
Socioeconomic status						
Low	600 (35.0)	176 (7.3)	**0.000009**	724 (30.2)	52 (2.2)	**0.002**
Middle	752 (31.3)	544 (22.7)		1,176 (49.0)	120 (5.0)	
High	116 (4.8)	212 (8.8)		320 (13.3)	8 (0.3)	
Total (%)	1,468 (61.2)	932 (38.8)		2,220 (92.5)	180 (7.5)	
Hx f/seizure						
Yes	260 (10.8)	308 (12.8)	**0.003**	528 (22.0)	40 (1.7)	0.975
No	1,156 (48.2)	676 (28.2)		1,704 (71.0)	128 (5.3)	
Total (%)	1,416 (59.0)	984 (41.0)		2,232 (93.0)	168 (7.0)	
NHIS						
Yes	90 (3.8)	22 (0.9)	**0.04**	96 (4.0)	16 (0.7)	**0.029**
No	1,307 (54.4)	981 (40.9)		2,136 (89.0)	152 (6.3)	
Total (%)	1,397 (58.2)	1,003 (41.8)		2,232 (93.0)	168 (7.0)	

grp, group; yrs, years; pos, position; pred, predominance; Hx, History; f, febrile; seizure, convulsion; NHIS, National Health Insurance Scheme.

Effect size estimates using Phi coefficient: knowledge, practice: age group- -, 0.101; gender-0.150, 0.110; number of siblings-0.220, -; child's position in the birth order-0.249, -; gender predominance- -, 0.106; nutritional status-0.295, -; socioeconomic status of the primary caregiver-0.280, 0.101; history of febrile covulsion-0.155, -; NHIS Status-0.107.

Bold values mean significant *P*-values (*P* < 0.05).

The differences in proportions for children's gender, socioeconomic status (SES) and national health insurance scheme (NHIS) status were statistically significant for both knowledge ad practice of childhood fever management with SES (for knowledge) having the largest effect size estimate (0.280). However, overall, the difference in proportion for nutritional status (for knowledge) have the largest effect size estimate (0.295) with NHIS (for practice) having the least effect size estimate (0.063).

### Based on the characteristics of the caregivers

Table 4 showed the characteristics of the caregivers that has statistically significant differences in proportions with regard to knowledge and practice of home-based childhood fever management.

**Table 4:** Comparison of the knowledge and practice of childhood fever management among the caregivers according to the characteristics of the caregivers (*N* = 2,400).

Characteristics	Knowledge inadequate	Adequate	*P* values	Practice inappropriate	Appropriate	*P* values
Age group (years)						
≤24	279 (11.6)	163 (6.8)	**0.04**	384 (16.0)	58 (2.4)	**0.00008**
25–34	666 (27.8)	566 (23.6)		1,148 (47.8)	84 (3.5)	
35–49	419 (17.5)	219 (9.1)		620 (25.8)	18 (0.8)	
≥50	29 (1.2)	59 (2.5)		80 (3.3)	8 (0.3)	
Total (%)	1,393 (58.0)	1,007 (42.0)		2,232 (93.0)	168 (7.0)	
Presence of second caregiver						
Yes	114 (4.8)	246 (10.3)	**0.00000009**	312 (13.0)	48 (2.0)	**0.0003**
No	1,341 (55.9)	699 (29.1)		1,920 (80.0)	120 (5.0)	
Total (%)	1,455 (60.6)	945 (39.4)		2,232 (93.0)	168 (7.0)	
Relationship between caregiver and child						
Parents	1,414 (58.9)	898 (37.4)	**0.000001**	2,152 (89.7)	160 (6.7)	0.682
Others	0 (0)	88 (3.7)		81 (3.4)	7 (0.3)	
Total (%)	1,414 (58.9)	986 (41.1)		2,233 (93.0)	167 (7.0)	
Marital status						
Married	1,406 (58.6)	866 (36.1)	**0.0000003**	2,120 (88.3)	152 (6.3)	0.125
Divorced	0 (0)	80 (3.3)		72 (3.0)	8 (0.3)	
Widowed	0 (0)	48 (2.0)		40 (1.7)	8 (0.3)	
Total (%)	1,406 (58.6)	994 (41.4)		2,232 (93.0)	168 (7.0)	
Type of current marriage *N *= 2,272						
Monogamy	894 (39.3)	754 (33.2)	**0.0000000002**	1,544 (68.0)	104 (4.6)	0.149
Polygamy	499 (22.0)	125 (5.5)		576 (25.4)	48 (2.1)	
Total (%)	1,393 (61.3)	879 (38.7)		2,120 (93.3)	152 (6.7)	
Position in the polygamy *N *= 624						
First	54 (8.7)	54 (8.7)	**0.001**	168 (26.9)	8 (1.3)	0.209
Second	435 (69.7)	54 (8.7)		384 (61.5)	40 (6.4)	
Third/Fourth	27 (4.3)	0 (0)		24 (3.8)	0 (0)	
Total (%)	516 (82.7)	108 (17.3)		576 (92.3)	48 (7.7)	

Effect size estimates using Phi coefficient:

Knowledge, Practice: Age Group-0.096, 0.134; Presence of Second Caregiver-0.284, 0.104; Relationship between Caregiver and Child-0.257, -; Marital Status-0.289, -; Type of Current Marital Status-0.371, -; Position in the Polygamy-0.399, -.

Bold values mean significant *P*-values (*P* < 0.05).

Only the differences in proportions for caregiver's age group, and the presence of a second caregiver were statistically significant for both knowledge ad practice of childhood fever management with presence of a second caregiver (for knowledge) having the largest effect size estimate (0.284). However, overall, the difference in proportion for position in polygamy (for knowledge) have the largest effect size estimate (0.399) with age group (for knowledge) having the least effect size estimate (0.063).

### Those who have adequate fever-related knowledge and engaged in appropriate practice

Only one out of every 100 caregivers (1.3%, 32/2,400) had both adequate fever-related knowledge and engaged in appropriate practices. Similarly, one out of six caregivers with adequate knowledge engaged in appropriate practices (Table 5).

**Table 5:** Comparison of knowledge and practice of childhood fever among the 2,400 caregivers.

Knowledge	Practice		Total
	Inappropriate Frequency (%)	Appropriate Frequency (%)	
Inadequate	1,266 (52.8)	136 (5.7)	1,402 (58.4)
Adequate	966 (40.3)	32 (1.3)	998 (41.6)
Total	2,232 (51.8)	168 (7.0)	2,400 (100)

### Determinants of knowledge and practice of childhood fever management

Only child's age group and presence of a primary caregiver determined both having adequate knowledge and engaging in appropriate practice of childhood fever management (Table 6). Specifically, primary caregivers [knowledge: odd ratio (OR): 2.81, 95% CI: 0.38; 5.68; practice: OR: 1.65, 95% CI: 0.09; 7.33] with a child/children aged ≤3 years (knowledge: OR: 7.03, 95% CI: 4.89; 9.67; practice: OR: 3.11, 95% CI: 1.27; 8.59) determined both the knowledge and practice of childhood fever management in a household (Table 6).

**Table 6:** Associations between characteristics of caregiver-children pairs with knowledge and practice of childhood fever management.

Characteristics	Knowledge			Practice		
	Odds ratio	95% CI	*P* values	Odds ratio	95% CI	*P* values
Child's age	7.030	4.890–9.670	0.003	3.110	1.270–8.590	0.007
Child's gender	–	–	–	0.395	0.242–0.647	0.000
SES	2.677	1.412–5.073	0.003	–	–	–
Hx of febrile convulsion	2.210	0.967–5.050	0.060	–	–	–
NHIS status	–	–	–	2.770	1.139–6.738	0.025
Presence of 2nd caregiver	2.810	0.380–5.680	0.041	1.650	0.090–7.330	0.020
Marital status	0.122	0.019–0.780	0.026	–	–	–

CI, confidence interval; SES, socioeconomic status; Hx, history; NHIS, National Health Insurance Scheme; 2nd: second.

All *p* values are significant (*p* ≤ 0.05).

## Discussion

Caregivers' adequate knowledge and appropriate practices were determined by having ≤3 year old children especially among primary caregivers aged 25–34 years in the household. Most caregivers exhibited fever phobic behaviors. More caregivers were knowledgeable than they were at engaging in evidence-based home healthcare delivery practices with very few of them possessing both qualities-adequate knowledge and appropriate practice. In addition, paracetamol was the most commonly used antipyretic medication prior to presentation with only a very few paracetamol-associated adverse events reported.

The youngest age group (≤3 years) constituted the highest number of febrile patients presenting at our study centers and this is consistent with the findings of Escalante and collaborators who assessed the antipyretic prescription pattern among 1,856 febrile children presenting at the health facilities of 13 developing countries spread across three WHO regions (42). This might have caused a lot of parental anxiety or fever phobia among the surveyed caregivers, especially for those in Africa and Asia due to their disproportionately high under-5 mortality rate (8, 42); especially in the presence of malnutrition (undernutrition) as was recorded in our study (43).

The level of fever phobia reported in our study is comparable to those from other studies from developing countries (12, 44). This comparability might stem from the fact that infectious diseases (often fever-inducing illnesses) are still a leading cause of morbidity and mortality among children and young adolescents in developing countries (7, 8). For instance, fever-inducing illnesses are the leading underlying etiologies of child morbidity and mortality in Nigeria (45); little wonder why caregivers witnessing febrile encounters would panic coupled with the perception of seeing themselves as a major line of defense against anything that threatens their children‘s health and wellbeing in general. Consequently, corroborating the finding that the presence of fever has significant effect on parents' fear of harms to their febrile children (10). In addition, the environments of developing countries have not completely transitioned from the historic one where infectious diseases held sway killing people in their millions to one found today (especially in developed countries where due to advancement in healthcare delivery and services occasioned by data-driven health planning and subsequent appropriate resource allocation) that kills reasonable number of people (18). Furthermore, HCPs should ensure they are knowledgeable about the concept, complications and treatment of fever as some parents have reported receiving conflicting fever-related information from different doctors for a single febrile episode of their children (21). Resistance to change among caregivers (perceived satisfactory outcomes using non-evidence based practices for addressing previous febrile encounters) and lack of international consensus on some basic aspects of CPGs is also reportedly culpable. These aspects include lack of consensus on the temperature that defines fever, preferred route for thermometry and temperature threshold to initiate antipyresis (19). Similarly, some parents expressed concerns over the inconsistencies (between HCPs' messages and their attitude towards their children's fever) they observed while some HCPs managed their children's fever, suggesting that the latter may understand the theoretical concept of fever they want to transmit but lack confidence in applying them (9). In addition, some parents have stated during most routine assessments of a febrile child, multiple HCPs will enquire about specific thermometer and body temperature readings (10). The above scenarios involving HCPs will confuse caregiver by reiterating the importance of temperature control (or numerical fever) and de-emphasizing focusing on the overall well-being of the febrile child; thereby perpetuating fever phobia among caregivers (10). Flowing from the above, HCPs should not only provide accurate and evidence-based fever-related information in a clear and easily understood language but should be empathetic and recognize the value of “maternal instincts” (9). From a cultural perspective, it is quite possible that due to cultural inertia (a seeming lag between current realities and historic events) the fear of fever might not be easily dispelled as childhood fever is ubiquitous (an experience most caregivers have encountered) and the phenomenon of fever phobia seemed to be transmitted from those whom the community members or the society perceived to be prestigious or successful (such as HCPs) (18). In addition, rectal thermometry seemed to be the preferred route for temperature measurement among caregivers in developed settings, while some cultures in developing settings may not be so embracing of it (10, 11, 18). Consequently, this aspect of cultural inertia may continue to affect the acceptance and uptake of currently recommended axillary and tympanic/auricular thermometry routes (16).

Another cause of parental anxiety is febrile convulsion (9, 16, 46). Like in previous studies, febrile seizure is one of the oft-cited consequences of untreated fever (complications of fever) among the caregivers surveyed in this study and having a child/ward with a history of febrile convulsion determined their knowledge of childhood fever even though this did not reach statistical significance (*p* = 0.061). Occurrence of febrile convulsion is often a life-changing experience for caregivers of febrile children and offers them the opportunity to receive relevant information from HCPs (9). The event may also lead to non-evidence based practices such as caregivers co-sleeping in the child's/wards' room or frequent temperature measurement during the episode (46) and overuse of antipyretics as an attempt to prevent subsequent febrile convulsion (9, 10, 16). This was corroborated by our study where the proportion of those who reported inappropriate practice was twice those who reported inadequate knowledge among caregivers whose children/wards have a history of febrile convulsion and the converse also applied: among caregivers who had encountered febrile convulsion those with adequate knowledge were 6 times more than those who reported adequate practice. This suggests that they may have the knowledge but febrile convulsion still make them anxious to the extent of engaging in non-recommended practices. Febrile convulsion is reportedly commoner among under-five children in developing countries (47, 48); and its prevalence may have been underreported due to cultural beliefs that it is caused by evil forces and witchcraft (thus better managed by a traditional medicine practitioner), stigma and discrimination (48). This belief is transmitted from generations to generations [with some HCPs culpable (46)] and may require targeted maternal education by taking advantage of antenatal and post natal visits of caregivers. It may also be necessary to launch a public health campaign in this regard with key evidence-based messages delivered in an easily understood language and format (46). As febrile convulsion is reportedly a risk factor for future epilepsy (47), sudden deaths in children (49) and post-traumatic stress disorder among caregivers (46); it would be appropriate for caregivers who have encountered febrile convulsion at home to present their children/wards at health facilities for thorough examination (to rule out sepsis and neurologic abnormality) and appropriate interventions (if necessary), counseling and education (including the distinction between simple and complex febrile convulsions and febrile status epilepticus) (16). In addition, presenting such children is highly imperative considering the proportion of children with moderate to very severe malnutrition (1 in 4 children) and low SES (1 in 3) in this study (16).

Like in previous studies, paracetamol was found to be the most frequently used antipyretic medication (next was ibuprofen) among the surveyed study participants (19, 36, 42), even though it may not be more effective than ibuprofen especially among under-fives (15, 35, 36, 50, 51) nor safer (15, 20, 52). The popularity and wide acceptance of paracetamol (over ibuprofen) may be related to its being in clinical practice much longer, recommended by all CPGs and expert consensus guide (15, 16, 23), for all age groups including preterm infants (15, 51). The safety concern of paracetamol is probably due to its toxic level being attained much sooner and causes more deaths than the supratherapeutic doses of ibuprofen (15). The reported efficacy edge of ibuprofen over paracetamol might be associated with the former having faster onset and greater symptomatic relief (leading to distress relieve being achieved faster and return to pre-morbid state attained earlier) and longer duration of antipyresis (leading to fewer doses being used and decreased chances of adverse events) (28, 53).

Paracetamol-associated adverse events (such as vomiting) was the most frequently reported adverse antipyretic use events among the caregivers surveyed in this study. No such report was linked with ibuprofen. This is consistent with the findings of Escalante (2,019) who conducted a facility-based survey among 2,117 febrile children and adolescents from 13 developing countries and reported antipyretic-related administration adverse events for paracetamol only (42). Similarly, the prevalence of the adverse event was comparable with that of our study (10/1,073 vs. 20/1,640-our study). In addition, undernutrition (malnutrition) and underweight are risk factors for paracetamol-associated adverse events (52). The occurrence of the adverse event may not be surprising considering that a quarter of the surveyed febrile children were malnourished. The consequent underweightedness (occasioned by undernutrition) might have increased the chances of the surveyed febrile children experiencing paracetamol toxicity (52).

Irrational use of antibiotics was prevalent (as all the antibiotics were used irrationally and only some of the antimalarials were used rationally) and antibiotic use-associated adverse events was the second most reported adverse events among the study participants. Fadare and collaborators have reported relatively easy access to antibacterial agents to be partly responsible for antibiotic overuse in developing countries (32) and one of the attendant consequences of this overuse, antimicrobial resistance (a global issue) has been reported in developing countries as well (21). In addition, antibiotic overuse might have indeed been responsible for the perceived antibiotic use-associated adverse events reported by the caregivers as Oshikoya and collaborators have reported antibiotics to be a major cause of adverse drug reactions in children and the potential for poor therapeutic outcome is also high (29, 32).

Measures that have been reported to promote safe home medication use among caregivers include caregivers' health literacy–appropriate verbal counseling strategies (such as teachback and showback in which caregivers demonstrate what has been learnt to the HCP), written caregiver education materials (including those with pictographic information) and provision of dosing-tool for liquid medication measurement (54).

The study setting (Nigeria) being a malaria endemic area justified the high prevalence of physician-diagnosed malaria cases recorded. However, only one out of every three febrile children (35.9% vs. 75.7%) diagnosed of malaria received an antimalarial at home (further worsened by some not receiving their complete doses due to perceived adverse events) before presentation leading to avoidable suffering of the poor care-recipients. This might have been avoided if caregivers were more knowledgeable about fever, use of rapid diagnostic test kits in community pharmacies that is available at subsidized rate. This might have prevented the visits (and its attendant wasteful use of resources and increased burden on healthcare services) of about one-half (43.7%) of the febrile children that were diagnosed of acute uncomplicated malaria in the centers. This needless visit due to poor knowledge of childhood fever has also been reported in developed settings (55) including the non-urgent use of ambulances (56).

Our findings regarding sub-optimal knowledge and inappropriate practices when caregivers self-manage their febrile children at home seemed to be a global issue (thus, becoming an issue of public health importance) as it has been reported by multi-country studies (10, 19, 30) and single-country studies conducted in developing (21, 57) and developed settings (14). However, the determinants of these gaps have not been well characterized especially in developing settings.

Being a married primary caregiver of at least middle SES and having child/children aged ≤3 years determined the possession of adequate knowledge among our study participants. Similarly, Arias and collaborators found that the younger the age of the child/children in a household the higher the likelihood of the caregiver having adequate knowledge of childhood fever management (14). This finding lend credence to the fact that younger aged children (especially the preschoolers) are more prone to fever-inducing illnesses and caregivers may have become knowledgeable in self-managing the febrile encounters overtime probably due to frequent encounters with healthcare services (13). They also reported higher household income (which could be likened to the middle-high SES prevalent in our study) a determinant of adequate knowledge. This could in part be explained by higher exposure to health-related information, more access to superior healthcare services, health literacy and self-efficacy as compared to caregivers of low SES or household income (14, 32). High level of maternal education and sufficient income have been correlated with adequate knowledge among Egyptian mothers of febrile children (57). However, no socioeconomic characteristics determined the childhood fever-related knowledge of Lebanese parents in a recent study (21). The reason for this is unclear but differences in study design (between this study and ours) might have been contributory.

Like in our study, Waly and Bakry (2022) found that more caregivers tended to be knowledgeable and yet lesser number of them engaged in recommended practices during febrile encounters of their children/wards (57). This may partly be due to both studies having comparable proportion of caregivers with fever phobic tendencies (68%-our study vs. 60%). Being a primary caregiver, having a female child aged ≤3 years with a national health insurance status determined engaging in evidence-based childhood fever management practices in our study. In contrast, younger maternal age, higher level of maternal education having sufficient income and fewer number of children determined appropriate childhood fever management practices among the Egyptian mothers (57). The reasons for the recorded differences are unclear but we suspect both cultural differences and differences in study design to be partly responsible. Cultural differences have been reported as a reason for the differences observed in the management practices of childhood fever by caregivers in the domiciliary contexts (9, 31, 57). Religion influences the cultural practices of a population; thus the Egyptian mothers were mostly full-time housewives; and likely less educated than our study population (as shown by their income level). Less education and insufficient income may necessitate seeking healthcare informally (from families and friends, etc) (57). The full-time housewives may have more spare time than the working class caregivers giving room for bonding and communal relationship that engender cultural inertia and transmission of information that might perpetuate inappropriate childhood fever management practices.

In order to bridge both the knowledge and practice gaps, we propose educational interventions involving the use of current CPGs on childhood fever that should target both HCPs and caregivers. Such interventions should use a mixed/blended approach that is culturally relevant and appropriate for a diverse education and socioeconomic levels and should involve demonstrations (such as face-to-face between trainers and trainees, and teachback (observation of the activities of trained caregivers) and computer simulation or audio-visuals or videos (58). The intervention should also target both knowledge and skill development/acquisition such as weight-based medication dosing (58). In addition, management of childhood fever should be a part of the undergraduate curriculum of those studying health-related courses (such as medicine, nursing and pharmacy) (30). In addition, faith-based organisations should be included as stakeholders during the development of any intervention, which should be freely available at all points of care, print and electronic media including social media (9). We also find it noteworthy to mention that an international summit be organized where researchers and other relevant stakeholders should deliberate on contentious issues regarding childhood fever management (such as what temperature constitutes fever, temperature threshold for initiating antipyresis, preferred route of temperature measurements, etc). Some researchers advocate for early presentation of febrile children by assessing delay in healthcare seeking while others advocate for home management of febrile children (especially those with minor illnesses). These schools of thoughts could lead to confusion and we hope such could be addressed through the summit.

This is probably the largest single-country, hospital based study from a developing setting that covered both rainy and dry seasons (the two major conditions of the Tropics), all fever-inducing illnesses among all pediatric age groups and assessed nutritional status of febrile children and then used a composite scoring system for knowledge and practices of childhood fever management. Despite these strengths, the study is limited by its cross-sectional nature but the large sample size may reduce any bias due to the study design.

## Conclusion

The knowledge and practice of the caregivers of febrile children surveyed were sub-optimal with being a primary caregiver and having a child/children aged ≤3 years being their determinants. The identified knowledge and practice gaps among the study participants underscore the dire need for targeted strategies aimed at improving the lot of the vulnerable care-recipients (febrile children) by educating their caregivers. In addition, following the world re-opening, there is a lot of international travels and ongoing transmission and mutation of COVID-19 making it imperative for these caregivers be taught how to identify fevers requiring their immediate attention and what to do, especially in developing settings (such as Nigeria) where infectious diseases are the leading cause of child mortality.

## Data Availability

The original contributions presented in the study are included in the article/**Supplementary Material**, further inquiries can be directed to the corresponding author/s.

## Supplementary material

Supplementary Table S1 Online questionnaire.
