# Supplementary material for: Determinants of caregiver's knowledge and practices regarding childhood fever management in a developing setting: a multi-centre cross-sectional assessment
Source: Front Pediatr. 2023 Aug 22;11:1119067. doi: 10.3389/fped.2023.1119067 (PMC10477664; doi:10.3389/fped.2023.1119067)
Supplement: Supplementary Table S1 — Online questionnaire. [file Table1.docx]

**QUESTIONNAIRE**

**SECTION A SOCIODEMOGRAPHIC DATA**

1a. Age of febrile child:….years; 1b. Gender: Male/Female; 1c. Weight:…Kg;

2a. Number of siblings:…; 2b. His/her position in the birth order: First/Second/Third/Other (specify)……..

3a. Number of male children:….; 3b. Number of female children:…..

4. History of febrile convulsion: Yes/No

5. Relationship of caregiver to the child: Parent/Grand parent/Other relations/Elder sibling/Friend/Nanny or Maid/Others (Specify)…………………….

6a. Gender of carer: Male/Female; 6b. Age of carer:….years

6c. Religion of caregiver:………; 6d. Tribe of carer:……..; 6e. Place of residence: Rural/Urban

7. Caregiver’s current marital status: separated/divorced/co-habiting/married/widowed/Single or Never married

8a. Type of current marriage: Monogamy/Polygamy; 9b. If polygamy, caregiver’s position in the union: First/Second/Third/Fourth

9. Caregiver’s educational level: No formal education/Primary school education/Secondary school education/Tertiary education

10. Caregiver’s occupation: Housewife/Trader/Farmer/Artisan/Business woman/Civil servant/Other (Specify)………….

11. Spouse’s educational status: No formal education/Primary school education/Secondary school education/Tertiary education

12. Spouse’s occupation: Trader/Farmer/Artisan/Business man/Civil servant/Unemployed/Other (Specify)………….

13. Beneficiary of National Health Insurance Scheme: Yes/No

14. Presence of more than one caregiver: Yes/No

15. Temperature of the febrile child at presentation in the study centre:…..ºC

**SECTION B KNOWLEDGE**

1. What caused your child to have fever (multiple responses allowed): Malaria/Teething/Diarrhoea/Bone pain crisis/Change of weather/Witch craft/Drugs/Convulsion/Typhoid/Anaemia (Shortage of blood)/Infection/Did not know
2. Physician’s diagnosis (from child’s records): Severe malaria with anaemia/Acute uncomplicated malaria/Cerebral malaria/Meningitis/Bronchopneumonia/Septicaemia/Diarrhoeal disease/Sickle cell anaemia/Otitis media/Others (Specify)…….
3. Are all fevers harmful: Yes/No
4. If yes, what are the harmful consequences of untreated fever (multiple responses are allowed): Dehydration /Seizure/ delirium or irrational talk / Coma / Blindness/Mental Retardation/Paralysis/Brain damage/Death/Nothing/Other (Specify)…………

**SECTION C PRACTICE**

During the fever episode, what did you do:

1a. Made sure the child had plenty of fluid to drink: Yes/No

1b. Covered the child up when he/she is shivering: Yes/No

1c. Changed the child’s cloth when he/she is sweating: Yes/No

1d. Woke the child up during the night to administer medication: Yes/No

1e. Apart from the above, what else did you do (multiple responses are allowed): Gave tea/Visited a patent medicine store/Visited a pharmacy/Visited a herbalist/Home treatment/Visited a health facility/Called or sent for a ‘nurse’ within the community/Called or sent for a friend/Called or sent for a relation/Others (Specify)………………………

If home treatment:

1f. What is the name of drugs/herbs used: Paracetamol/Ibuprofen/Aspirin/Sulphadoxine-pyrimethamine/Chloroquine/ACT/Co-trimoxazole/Ampicillin-cloxacillin/Co-amoxiclav/Herbs/ORS/Others (Specify)……………../Do not know

2. Where did you get the drug/herb that you used: Provision shop/Patent medicine vendor/Pharmacy/Health facility/Relatives/Friends/Remnant of the previously used drug stored at home/Herbalist/Others (Specify)………………………

3. Where did you get the information about the dose of drug/herb administered to the child: Provision shop owner/Patent medicine seller/Pharmacist/Herbalist/Doctor or Nurse in a health facility/Previous encounter with a doctor or nurse in a health facility/Previous experience/Reading the package leaflet of the medicine/Friends/Family member/Internet/Social media/Electronic media/Print media/Self or guessed/Spouse/Others (Specify)………………………

4. What additional factor did you consider when administering the dose of the antipyretic drug (Multiple response are allowed): Age/Weight/Height/Temperature/Illness severity/Lethargy/Previous experience

5. What did you use to determine the dose of the antipyretic drug: Tablespoon/Teaspoon/The drug manufacturer’s measuring spoon or cup or dropper/The cap or lid of the medicine bottle or plastic/Another drug manufacturer’s measuring cup or cap or dropper/Others (Specify)………….

6. How many times did you administer the antipyretic drug within the last 24 hours: 3/4/5/6/7 times

7. What did you do to ensure the dosage interval was accurate (multiple responses allowed): Alarms/Wrist watch/Wall clock/Time on mobile phones/Muslims’ prayer times/Sun’s or moon’s position/Other family members reminding the carer/Others (Specify)………………………..

8a. Did the child’s body temperature (hotness) ever remained the same for up to 2 hours even after administering the medicine: Yes/No

8b. If yes, what did you do (multiple responses are allowed): Changed the dosage form/Changed to another drug but in the same therapeutic category as the first/Changed from one therapeutic category to another/Added a different dosage form of the same drug/Added another drug from the same therapeutic category as the first/Added a drug from a different therapeutic category/Added external cooling measures or supportive measures/Went to a healthcare facility as soon as possible/Waited or did nothing

9a. Did the child ever vomited after the medicine was administered: Yes/No

9b. If yes, what did you do (multiple responses allowed): Gave a smaller dose of the same medicine/Gave the same dose of the medicine again/Gave a higher dose of the same medicine/ Changed the dosage form/Changed to another drug from the same therapeutic category/Changed from one therapeutic category to another/Added a different dosage form of the same drug/Added a drug from a different therapeutic category/Added external cooling measures or supportive measures/Went to a healthcare facility as soon as possible/Waited or did nothing

10. Did you ever forget to administer the medicine at any pre-arranged time: Yes/No

10b. If yes, what did you do (multiple responses allowed): Gave the medication as soon as he/she remembered/Provided next dose before its time/Gave forgotten dose with next dose together (doubled the dose)/Omitted the forgotten dose and waited for the next pre-arranged time to administer the next dose/Called a doctor/Called a nurse/Called a pharmacist/Others (Specify)…………

11. What did you do to ensure the child takes the medicine whenever he/she is required to (multiple responses allowed): Refer to medication as a honey or candy/Pinch child's nose to force the child to swallow the medication/Restrained and forced the child to take the medication/Asked a loved person to administer medications to the child/Rewarded and praised the child to take the medication/Threatened the child if he/she refuses the medication/Others (Specify)…………………

12. How did you store the medication after each use (multiple responses allowed): Left in its original pack/In the refrigerator/On the refrigerator/Shelf/First-aid box/In locked cabinet/Others (Specify)……..

13. Apart from using antipyretic medicine, what else did you do (multiple responses allowed): Applied cold compresses/Applied ice packs/Gave warm bath/Wrapped the child with a blanket/Sponging with warm water/Sponging with cold water/Increased the number of clothes the child is wearing/Reduced the number of clothes/Completely undressed the child/Increased food intake/Decreased food intake/Increased fluid intake/Decreased fluid intake/Waited/Others (Specify)………………………..

14a. Did the child experience any side effect while taken the medications: Yes/No

14b. If yes, which drug was suspected? Paracetamol/Ibuprofen/Aspirin/Sulphadoxine-pyrimethamine/Chloroquine/ACT/Co-trimoxazole/Ampicillin-cloxacillin/Co-amoxiclav/Herbs/Others (Specify)……………../Do not know

14c. The suspected side effect is: Vomited each time the particular medication/herb was taken/Had diarrhea each time the particular medication/herb was taken/Loss of appetite/Stomach ache/Dizziness/Others (Specify)……………………..

14d. What did you do upon noticing the side effect: Stopped using the suspected medication /Gave a smaller dose of the same medicine/Gave the same dose of the medicine again/Gave a higher dose of the same medicine/Changed the dosage form/changed from one therapeutic category to another/Added a different dosage form of the same drug/Added a drug from a different therapeutic category/Added external cooling measures or supportive measures/Went to a healthcare facility as soon as possible/Waited or did nothing
